# Highly sensitive inference of time-delayed gene regulation by network deconvolution

**DOI:** 10.1186/1752-0509-8-S4-S6

**Published:** 2014-12-08

**Authors:** Haifen Chen, Piyushkumar A Mundra, Li Na Zhao, Feng Lin, Jie Zheng

**Affiliations:** 1 School of Computer Engineering, Nanyang Technological University, 50 Nanyang Avenue, 639798 Singapore, Singapore; 2 Metabolomics Laboratory, Baker IDI Heart and Diabetes Institute, 3004 Melbourne, Australia; 3 Genome Institute of Singapore, A*STAR, Biopolis, 138672 Singapore, Singapore

**Keywords:** GRN inference, time delay, cross-correlation, network deconvolution

## Abstract

**Background:**

Gene regulatory network (GRN) is a fundamental topic in systems biology. The dynamics of GRN can shed light on the cellular processes, which facilitates the understanding of the mechanisms of diseases when the processes are dysregulated. Accurate reconstruction of GRN could also provide guidelines for experimental biologists. Therefore, inferring gene regulatory networks from high-throughput gene expression data is a central problem in systems biology. However, due to the inherent complexity of gene regulation, noise in measuring the data and the short length of time-series data, it is very challenging to reconstruct accurate GRNs. On the other hand, a better understanding into gene regulation could help to improve the performance of GRN inference. Time delay is one of the most important characteristics of gene regulation. By incorporating the information of time delays, we can achieve more accurate inference of GRN.

**Results:**

In this paper, we propose a method to infer time-delayed gene regulation based on cross-correlation and network deconvolution (ND). First, we employ cross-correlation to obtain the probable time delays for the interactions between each target gene and its potential regulators. Then based on the inferred delays, the technique of ND is applied to identify direct interactions between the target gene and its regulators. Experiments on real-life gene expression datasets show that our method achieves overall better performance than existing methods for inferring time-delayed GRNs.

**Conclusion:**

By taking into account the time delays among gene interactions, our method is able to infer GRN more accurately. The effectiveness of our method has been shown by the experiments on three real-life gene expression datasets of yeast. Compared with other existing methods which were designed for learning time-delayed GRN, our method has significantly higher sensitivity without much reduction of specificity.

## Background

The inference of a gene regulatory network (GRN) is a vital step in understanding many biological systems in detail. However, the inference of GRN is known to be challenging due to several facts: (1) gene regulation is inherently complicated, (2) the measurements of gene expression levels are usually noisy, (3) the datasets for GRN inference are often incomplete, (4) time-series gene expression datasets have short time series compared to the number of genes measured. Generally, a GRN is inferred using machine learning algorithms on a time-series gene-expression dataset. Given the time-series data, the gene regulation could be inferred in two ways: one is assuming instantaneous or first order regulation, and the other is considering higher order regulation. In many cases, a gene regulates the expression of another gene by its products (RNAs or proteins). Since it takes time to generate those products and different processes (e.g. transcription, translation) need different amounts of time, time-delayed regulation is ubiquitous in cellular processes. Thus, inferring time-delayed gene interactions is essential to accurately reconstructing GRN.

The problem of inferring higher-order time delays is challenging, due to the tremendous search space when the numbers of time lags are unknown. For the *r*-th order system with totally *T *time points in the dataset, the available numbers of time points for inference reduce to T-*r*. This poses a serious computational challenge resulting in more false predictions.

While many methods have been introduced to reconstruct first-order gene regulation (e.g. DBN-MCMC [1-3], dynamic RandomForest [4]), there are only a few methods for inferring time-delayed GRN. In 2010, a dynamic version of ARACNE (Algorithm for the Reconstruction of Accurate Cellular Networks) was introduced to infer time-delayed dependencies among genes [5]. Their method, called TimeDelay ARACNE (or TD-ARACNE), is able to reconstruct time-delayed dependencies effectively. In 2012, Morshed *et al*. proposed a framework to infer instantaneous and time-delayed genetic interactions at the same time [6]. Their approach was shown to outperform some existing methods such as TD-ARACNE and BANJO. In 2013, Li *et al*. presented a method to infer high-order gene regulation, named MMHODBN (max-min high-order dynamic Bayesian network) [7]. MMHODBN is a hybrid Bayesian network method, which incudes two steps: first it learns the skeleton (i.e. an undirected network) of GRN using constraint-based Bayesian learning (Spirtes *et al*., 2001); then it performs a search-and-score technique to orient the edges in the skeleton of GRN. It was shown that MMHODBN was able to learn high-order gene interactions effectively. Mundra *et al*. proposed a method for inferring time-delayed GRN based on cross-correlation and LASSO [8]. This method has been tested on real-life yeast pathways in G1 phase to show its effectiveness in identifying time-delayed regulation among genes. Despite all those efforts, the performance of inferring time-delayed genetic regulation is yet to be further improved.

In this paper, we propose a simple yet effective and efficient method to tackle the challenges of inferring high-order time-delayed gene regulation. Using cross-correlation [9,10] and data manipulations, we first determine the probable time lags and then use the algorithm of network deconvolution (ND) [11] to infer the time-delayed GRN. ND is a technique to identify direct dependencies in an observed network (e.g. correlation-based network) which contains both direct and indirect interactions. By assuming that the indirect edges could be estimated from the products of direct edges and the observed network is the sum of the direct and indirect edges, ND can recover the direct network from the observed network through the process of deconvolution. However, the authors of ND methods have not considered time delays, i.e. they assume all direct interactions take equal time, which is unlikely in the real biological systems. Our method integrates time delay inference and adjustment into the ND approach, to further increase its power. Running on three real-life datasets of yeast, the proposed method achieves better performance than existing methods.

## Results and discussion

We proposed a method to infer time-delayed gene regulation based on cross-correlation and network deconvolution. We first identified the probable time delays for the interactions between each target gene and its potential regulators, using cross-correlation[9,10]. Then, we adapted the algorithm of network deconvolution [11] to infer time-delayed genetic interactions. Network deconvolution has been shown to be very promising in learning gene regulation [11]. However, ND does not consider time delays, which are essential in gene interactions. Besides, the network inferred by ND is indirected (i.e. without directions in edges). Here we introduced time delays into ND to enhance its strength in GRN inference. Based on the time delays identified with cross-correlation, we aligned the samples and calculated correlations of genes using the aligned samples. Then we applied ND to the correlation matrix and identified the direct interactions between the target gene and its regulators. The network inferred by our method is directed and includes time delays. We have evaluated the performance of our method on three gene expression datasets, described as follows.

### Benchmark networks

**Figure 1:**
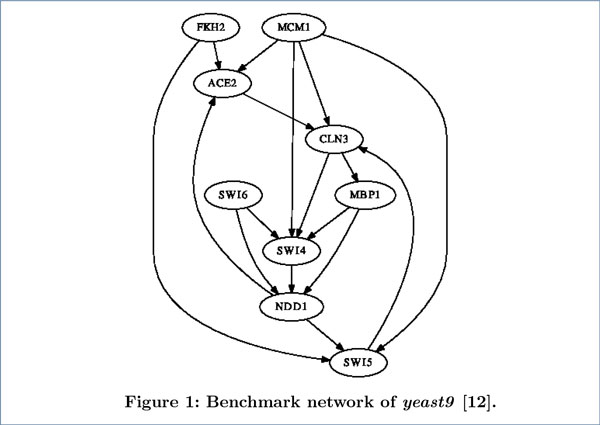
**Benchmark network of yeast9**. This benchmark network is from [12]. All edges were detected by biological experiments.

**Figure 2:**
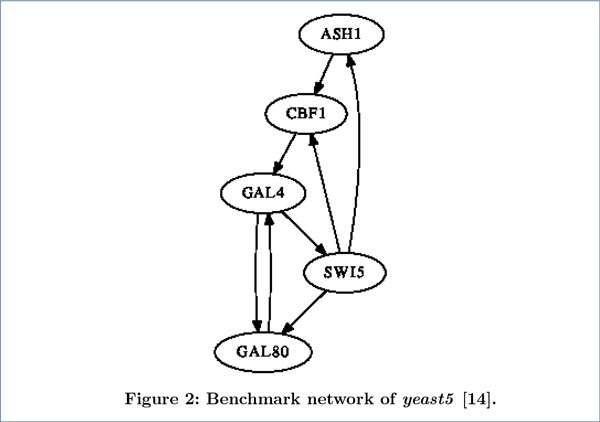
**Benchmark network of yeast5**. This benchmark network is from [14]. All edges are with experimental supports.

We evaluated the performance of our method on two real-life benchmark networks and compared with other related methods. One benchmark network (see Figure 1) is experimentally identified in *Saccharomyces cerevisiae *(yeast) cell cycle [12]. This network consists of nine genes (ACE2, CLN3, FKH2, MBP1, MCM1, NDD1, SWI4, SWI5 and SWI6). The real expression data (denoted as *yeast9 *) of genes in this network were taken from Spellman [13], which consists of the transcription expression data of yeast cell cycle. We extracted the time-series data from cdc-15 cell cycle arrest which contains 24 equally distributed time points. The other benchmark network (see Figure 2) is a five-gene network in yeast, from the experiment of *in vivo *reverse-engineering and modeling assessment (IRMA) [14]. This network was carefully constructed to include the interactions among five genes (CBF1, GAL4, SWI5, GAL80 and ASH1) in *Saccharomyces cerevisiae *and made sure that the influence from endogenous genes is negligible. Two datasets of gene expression were measured for this network. One dataset was obtained when the cell culture was shifted from glucose to galactose. This dataset was named "switch-on" because the network would be triggered by galactose. The other dataset was named "switchoff" since it is obtained by shifting the cell culture from galactose to glucose. The "switch-on" dataset (denoted as *yeast5on*) consists of 16 equally distributed time points, and the "switch-off" dataset (denoted as *yeast5off *) contains 21 equally distributed time points.

### Experimental results

There are two parameters in time-delayed ND (i.e. the proposed method). One is the threshold *θ *(0 *≤ θ ≤ *1) for the matrix output by ND. Since this matrix of ND is a weighted matrix where each entry represents the strength of interaction between the corresponding gene pair, we need to set up a threshold to obtain a connectivity matrix from this weighted matrix. Then we can compare the connectivity matrix with the benchmark network and calculate performance metrics such as *sensitivity *and *F-measure*. In all the following experiments (except the ROC cureves), we set the threshold *θ *to a moderate value (i.e. 0.5). The other parameter is the maximum time lag *r*. Since a large *r *would lead to a small number of samples available to infergene interactions, we usually set *r ≤ *5. We have done experiments with *r *taking values from 2 to 5. The results of comparing to other methods are similar (data not shown). Here we only show the results with *r *= 5. In addition, we show the results with *r *= 3 for the *yeast5off *dataset to compare.

**Figure 3:**
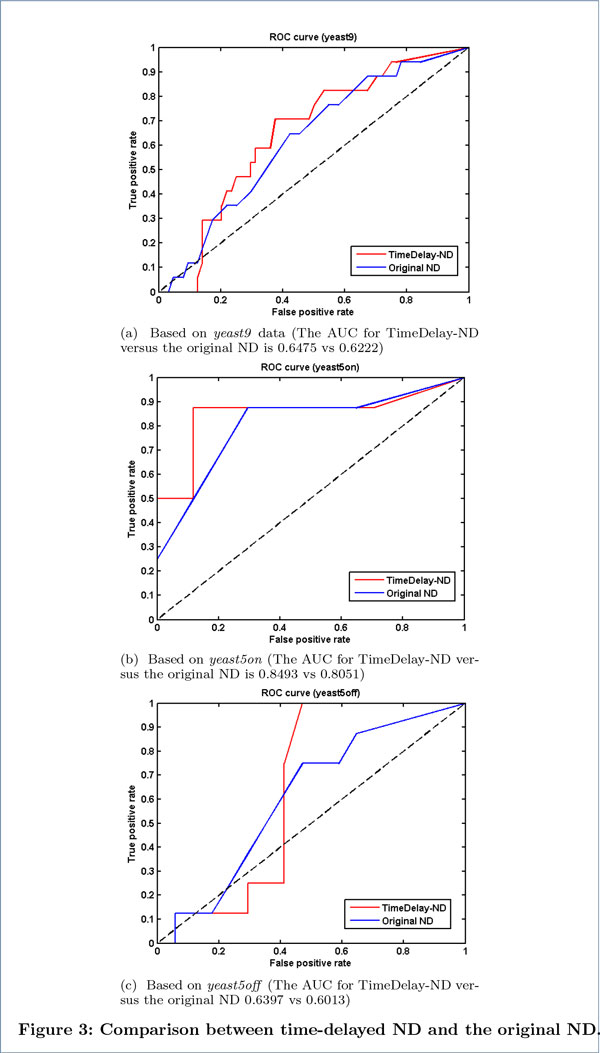
**Comparison between time-delayed ND and the original ND**. The ROC curves are drawn by changing the parameter *θ *from 0 to 1. The maximum time lag *r *in time-delayed ND is set as 5 for *yeast9 *and *yeast5on*, and 3 for *yeast5off*. The overall performance of time-delayed ND and the original ND is compared based on the AUC values calculated from the ROC curves.

First we compared time-delayed ND (i.e. the proposed method) with the original ND (no delay). By evenly changing the values of the threshold *θ *(0 *≤ θ ≤ *1), we can generate a set of performance metrics. Then a receiver operating characteristic (ROC) curve could be plotted to show the overall performance of a method with the results obtained from the whole set of different thresholds. The comparison between time-delayed ND with the original ND (no-delay ND) is shown in Figure 3, where "False positive rate (FPR)" is defined as "1*−specificity *", and "True positive rate (TPR)" is equal to "*sensitivity *". Overall, time-delayed ND outperforms the original ND in that the AUC of the former is larger than the latter for all three datasets. Moreover, when TPR (i.e. *sensitivity *) is high (e.g. *≥ *0.5), the FPR of time-delayed ND is lower than the original ND most of the time, which means time-delayed ND has a higher *specificity*. These results show that the accuracy of GRN inference is improved by taking into account the time delays among gene interactions.

To compare time-delayed ND with other existing methods which are designed for inferring time-delayed gene regulation, we apply these methods on the same dataset and compare their performance using *sensitivity, precision*, and *F-measure*. There are three existing methods available for our comparisons, namely time-delayed ARACNE (TD-ARACNE) [5], Xcorr+LASSO [8,15], and MMHODBN [7].

**Figure 4:**
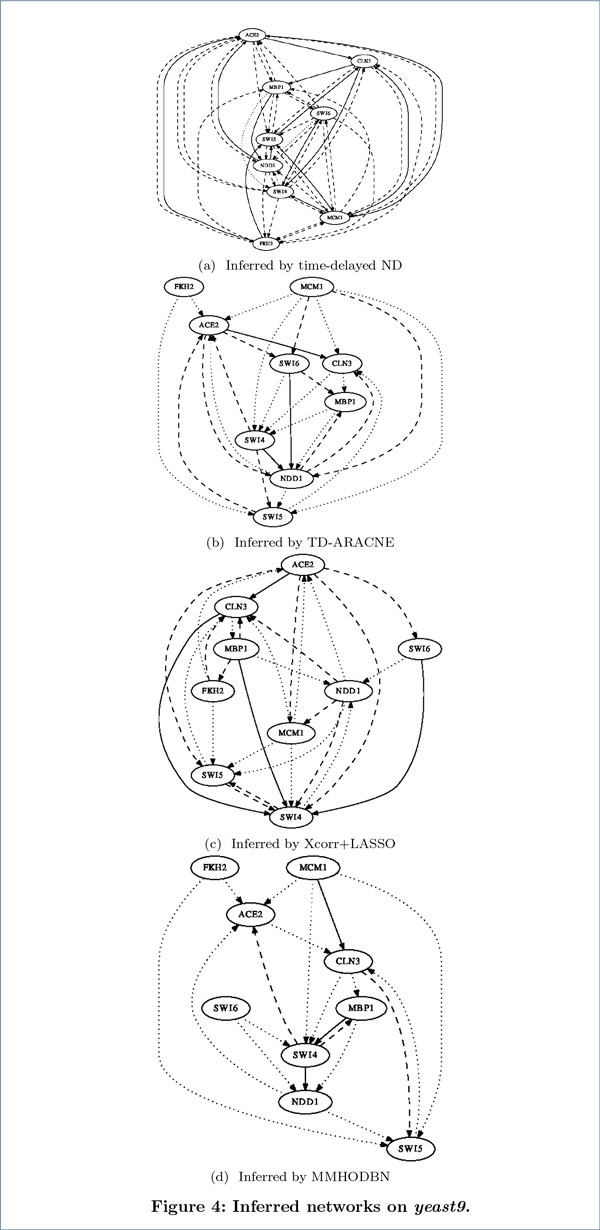
**Inferred networks on yeast9**. In all inferred networks, solid lines, dashed lines and dot lines denote true positives, false positives and false negatives respectively (likewise for Figure 5 and Figure 6).

**Figure 5:**
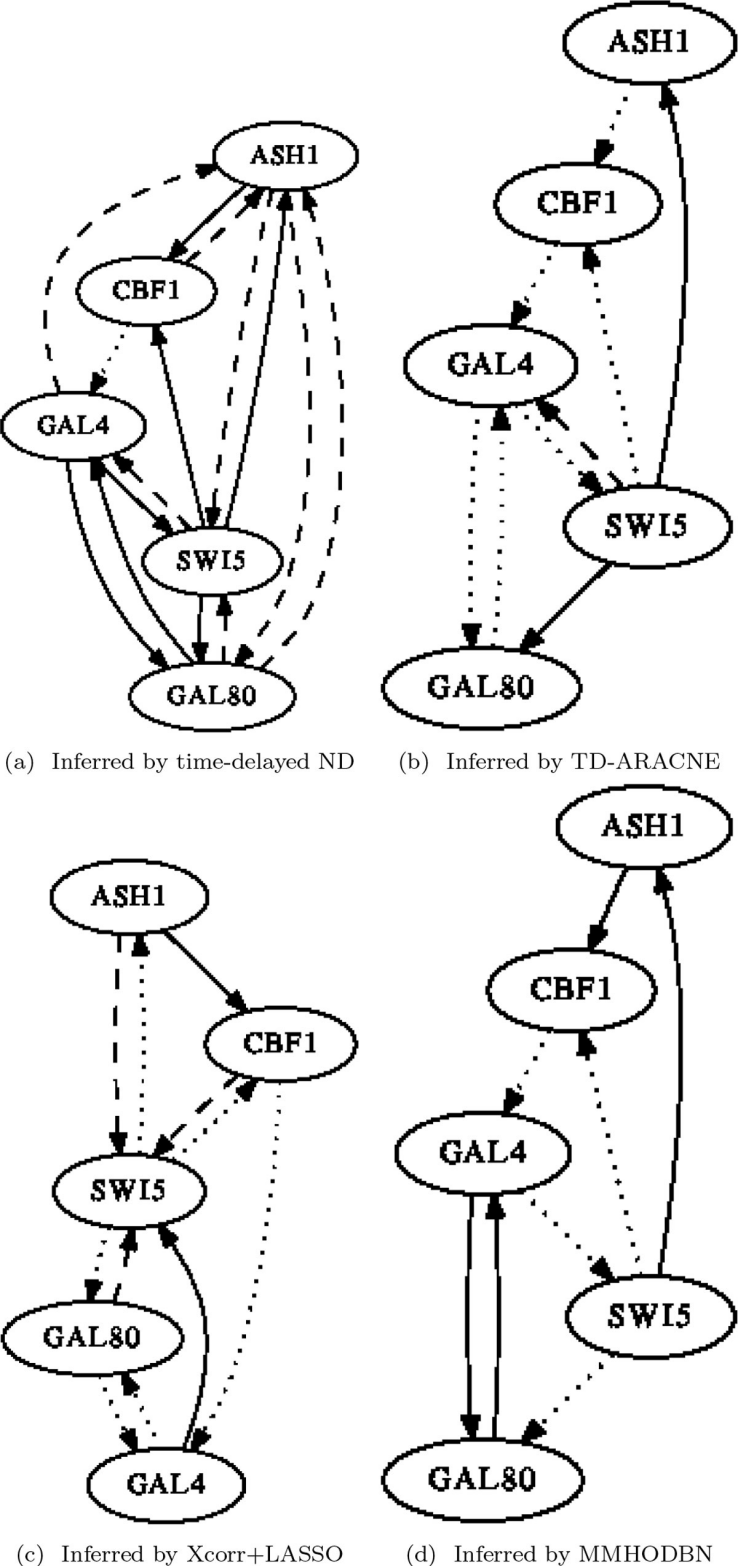
**Inferred networks on yeast5on**.

**Figure 6:**
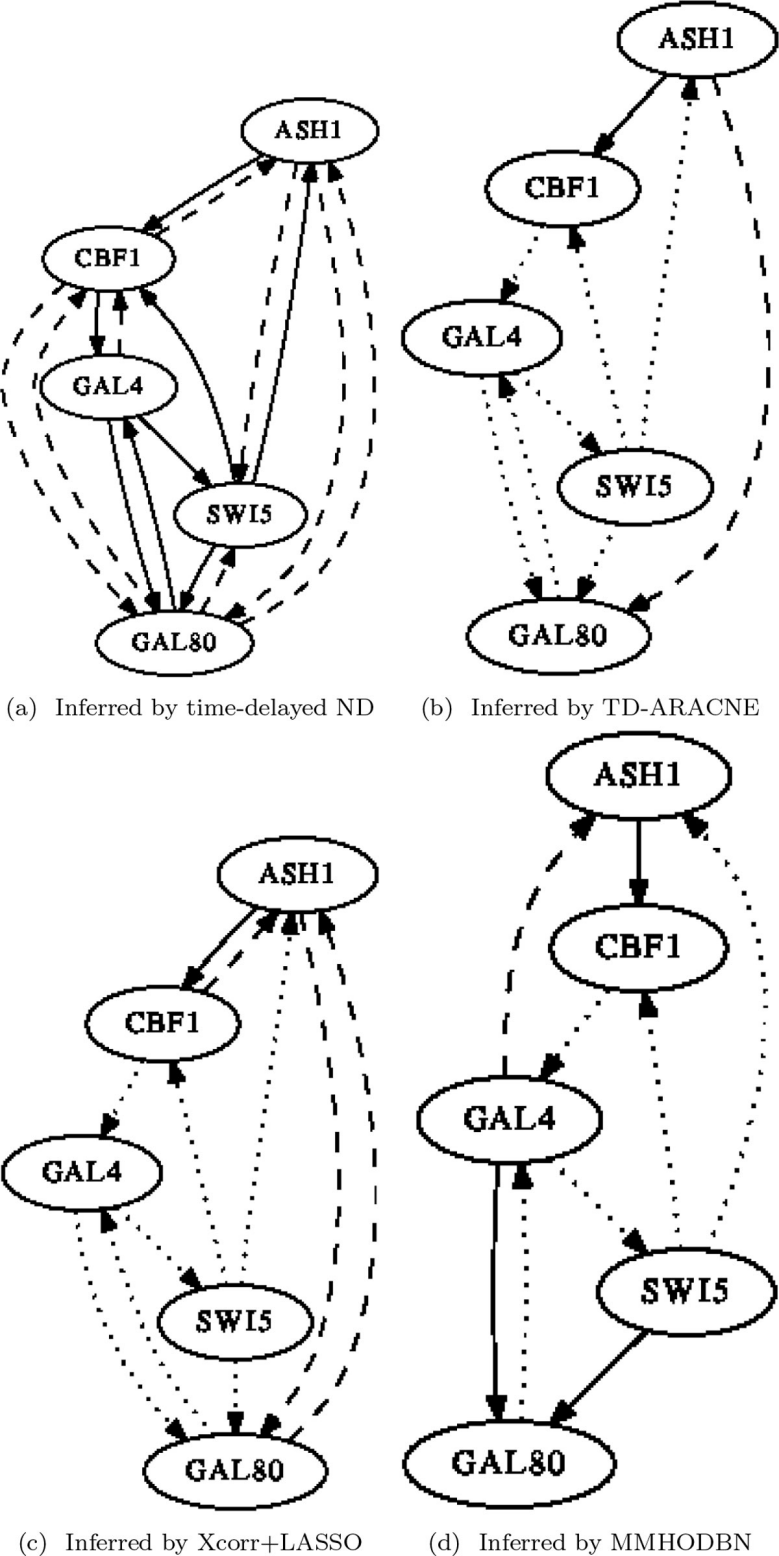
**Inferred networks on yeast5off**.

We first carried out experiments on the *yeast9 *dataset. The inferred networks by the four methods are presented in Figure 4, where solid lines, dashed lines and dot lines denote true positives, false positives and false negatives respectively (likewise for Figure 5 and Figure 6). The performance comparison between our method with the other three methods in Table 1 shows that time-delayed ND has significantly higher *F-measure *than all other methods, which means our method can infer time-delayed GRN more accurately. Although the *precision *of MMHODBN is higher than time-delayed ND, its *sensitivity *is lower. We also performed experiments on another benchmark network (shown in Figure 2), which contains two datasets: *yeast5on *and *yeast5off*. The inferred networks by the four methods on the *yeast5on *and *yeast5off *are presented in Figure 5 and Figure 6 respectively. Table 2 and Table 3 show the performance comparison of the four methods on *yeast5on *and *yeast5off *respectively. In Table 2 the *F-measure *of time-delayed ND ranks the second place among the four methods. Although the *F-measure *of time-delayed ND is slightly lower than MMHODBN, it still has higher *sensitivity *than the latter. For *yeast5off *dataset, as shown in Table 3 the *F-measure *of time-delayed ND is again the highest among the four methods.

**Table 1:** Comparison based on *yeast9 *dataset.

	Precision	Sensitivity	F-measure
**time-delayed ND**	0.2917	0.8235	0.4308
TD-ARACNE	0.2307	0.1764	0.2000
Xcorr+LASSO	0.2500	0.2353	0.2424
MMHODBN	0.5000	0.1765	0.2609

**Table 2:** Comparison based on *yeast5on *dataset.

	Precision	Sensitivity	F-measure
**time-delayed ND**	0.5000	0.8750	0.6364
TD-ARACNE	0.6667	0.2500	0.3636
Xcorr+LASSO	0.4000	0.2500	0.3077
MMHODBN	1.000	0.5000	0.6667

**Table 3:** Comparison based on *yeast5off *dataset.

	Precision	Sensitivity	F-measure
**time-delayed ND**	0.5000	1.000	0.6667
TD-ARACNE	0.5	0.125	0.2000
Xcorr+LASSO	0.2500	0.1250	0.1667
MMHODBN	0.6667	0.2500	0.3636

As the results of the three experiments suggested, time-delayed ND has a high *sensitivity *in detecting time-delayed gene interactions, which yields a better performance (in terms of *F-measure*) than other methods.

### Discussion

The network deconvolution algorithm is a nonlinear filter which could be applied to any symmetric (and some asymmetric) network matrix to filter out the indirect edges. Using correlation in the original method, only an undirected network could be inferred. In the proposed method, the correlation is calculated between each target gene and the rest of genes. The correlation matrix also includes correlations between all the other genes using the time samples aligned based on the inferred time delays between these genes and the target genes (see Algorithms 1 and 2). ND is then applied to such a correlation matrix, i.e., the filter for indirect edges is determined using correlation not only between a target gene and the rest of genes but also between the rest of genes correlations. This step helps in the determination of regulatory direction while considering redundancy in the possible regulators.

In the proposed method, the networks are inferred with a *priori *determined maximum time lag *r *and a threshold *θ*. Given the short time series, increasing maximum possible time lag will reduce the available number of time points to compute the correlations. This may ultimately result in an increase in the numbers of false predictions. Hence, we have restricted the maximum possible time lag to 5 in our experiments. An increase in the value of *θ *means increasing the cut-off to infer a regulatory edge. Generally, this increase will result in only partial recovery of true positive edges. Hence in our experiments, we have kept the value of *θ *to a moderate level so that most of the true positives edges could be recovered while keeping the false positives in check.

## Conclusions

In this paper, we proposed a method named time-delayed ND to infer time-delayed gene interactions based on cross-correlation and network deconvolution. We first infer the probable time delays for the interactions between each target gene and its potential regulators, using cross-correlation. Then based on the inferred time delays, we align the time samples for each target gene. After that, we employ the algorithm of network deconvolution to identify direct interactions between the target gene and its regulators. The performance of time-delayed ND has been evaluated on three real-life gene expression datasets. Compared with three other methods for inferring time-delayed GRNs, our method achieved overall better performance in the inference of time-delayed GRN.

## Methods

The time-delayed gene regulatory network is inferred using a time-series gene expression data. Let *X_T ×N _*= (*x*_1_*, x*_2_*, ..., x_N _*) be a time-series gene expression dataset where *N *is the number of genes and *T *is the number of time samples. Let *x_t,i _*denote the expression level of the *i*-th gene at time *t*. Then *x_i _*= (*x*_1*,i*_*, x*_2*,i*_*, ..., x_T ,i_*)^T^, where 1 *≤ i ≤ N *denotes the expression profile of the *i*-th gene across *T *time points.

To infer regulatory interactions among genes, the most straightforward way is by using correlations. However, there are two major issues about correlations: (1) time-delayed regulation is not likely to be inferred by simple correlations; (2) the relationships based on correlations are not direct and would suffer from a large number of false positive predictions. The two issues can be coped with by cross-correlation and network deconvolution respectively, which are described in the following two sections.

### Cross-correlation

To infer a time-delayed regulation between the *i*-th gene and *j*-th gene, we need to determine the number of time lags first. This could be achieved by applying cross-correlation [9,10] on the expression profiles of these two genes. The lag that gives the maximum absolute cross-correlation is the most likely time lag.

For two real continuous functions *x *and *y*, the cross-correlation *ϕ_xy _*is defined as [9]:

(1) $$\phi_{xy}\left(\tau \right)=\int_{-\infty }^{\infty }x\left(t-\tau \right)y\left(t\right)dt.$$

The cross-correlation *ϕ_xy_*(*τ*) can be obtained by inverse Fourier transform (iFT) as follows:

(2) $$\phi_{xy}=iFT\left[\Phi_{xy}\left(\tau \right)\right]=iFT\left[X^{*}\left(f\right)Y\left(f\right)\right].$$

where Φ*_xy _, X *and *Y *are *ϕ_xy_, x *and *y *in frequency domain respectively, *X*^∗ ^is the conjugate of *X*. The derivation of Eq. 2 is shown in the endnote of this paper. ^1 ^The cross-correlation could also be obtained in another way as follows. Let *r *be the maximum length of time delays, and *τ *be one possible lag in a time dalay, i.e. *τ *= 0, 1, 2*, ..., r*. For the *i*-th gene and the *j*-th gene (with expression profiles denoted as *x_i _*and *x_j _*respectively), the unbiased cross-correlation is defined as in [9,8]:

(3) $$C\left(x_{i},x_{j},\tau \right)=\frac{1}{T-|\tau |}\overset{T-\tau -1}{\underset{t=1}{\sum }}x_{t+\tau ,i}x_{t,j},\;\;\text{where}\;\tau =0,1,2,\ldots ,r.$$

Here, *x_i _*and *x_j _*are the expression profiles. Note that we normalized the gene expression data for each gene to have "zero" mean and "one" standard deviation before calculating the cross-correlation.

In this paper, we utilize the Matlab function *xcorr *which adopts the previous way (i.e. Eq. 2) to calculate cross-correlations. For each target gene, cross-correlation is calculated between this gene and all the other genes. From the maximum possible *r *time lags, we identify the time-delayed *τ *which corresponds to the maximum absolute values of *C*(*x_i_, x_j _, τ *). This time delay is denoted by *l_ij _*and it represents the probable time lag of regulation between the *i*-th gene and the *j*-th gene.

### Network deconvolution

After determining the probable time lag for each gene pair, we can proceed to determine the possible regulators for each target gene from the rest genes in the dataset. As mentioned above, using correlations is a natural way to identify interactions among genes; but such approach may suffer from a large number of false positive predictions. The main reason is that most correlations represent indirect dependencies instead of direct dependencies. A direct dependency between two variables mean that the interaction between the two variables does not depend on any intermedium. On the other hand, the indirect dependency is caused by direct dependencies through some intermediate nodes. For example, if *A *regulates *B *and *B *regulates *C*, even though there is no direct relationship between *A *and *C*, the correlation between *A *and *C *could be high because there is an intermediate node *B *between them. Network deconvolution (ND) [11] is a technique to infer direct dependencies among variables. Let us use a matrix to represent a network. Starting from the matrix of correlations (or other similarity metrics) which could include both direct and indirect dependencies, ND is able to filter out the indirect dependencies through a process called network deconvolution.

Let *S_o _*be the observed network (i.e. the correlation matrix), and *S_d _*be true network with only direct edges. ND assumes that the indirect edges could be derived from the product of direct edges, and the observed network *S_o _*is the sum of the direct and indirect edges, as follows.

(4) $$S_{o}=S_{d}+S_{d}^{2}+S_{d}^{3}+S_{d}^{4}+\cdots \;.$$

The task of obtaining *S_d _*from *S_o _*seems intractable because of the infinite sum. However, by using the closed form solution of infinite Taylor series, we can have

(5) $$S_{o}=S_{d}\left(I+S_{d}^{2}+S_{d}^{3}+S_{d}^{4}+\cdots \;\right)=S_{d}{\left(I-S_{d}\right)}^{-1},$$

where *I *is the identity matrix. Through simple transformation, we have

(6) $$S_{d}=S_{o}{\left(I+S_{o}\right)}^{-1}.$$

It has been shown in [11] that all symmetric matrices and some asymmetry matrices (*S_o_*) can be decomposed into their eigenvectors and eigenvalues. Let *U *be the matrix of eigenvectors, and Σ*_o _*be the diagonal matrix of eigenvalues of *S_o_*. Then *S_o _*= *U*Σ*_o_U*^−1^. Similarly, we have *S_d _*= *U*Σ*_d_U*^−1^. Let $\lambda_{i}^{o}$ be the *i *th eigenvalue of *S_o_*, and $\lambda_{i}^{d}$ be the *i *th eigenvalue of *S_d_*. Through Eq. (6), we have

(7) $$\lambda_{i}^{d}=\frac{\lambda_{i}^{o}}{1+\lambda_{i}^{o}}.$$

To guarantee the convergence of Eq. 4, which requires that the largest absolute eigenvalue of *S_d _*is less than one, the authors of ND introduced a scaling factor *α*. Then Eq. 7 is changed to

(8) $$\lambda_{i}^{d}=\frac{\lambda_{i}^{o}}{\frac{1}{\alpha }+\lambda_{i}^{o}}.$$

In our code, the scaled version (i.e. Eq. 8) of ND was implemented. We used the default value for the parameter of *α*.

Experimental results in [11] show that when their assumptions hold that the indirect edges can be derived from the product of direct edges and the observed network is the sum of direct and indirect edges, the method can remove all indirect edges and recover all direct edges; even when the assumptions do not hold, it still can infer most of direct interactions, as shown by simulation experiments on various network structures. For more details about ND, please refer to [11].

### Time-delayed ND

**Figure 7:**
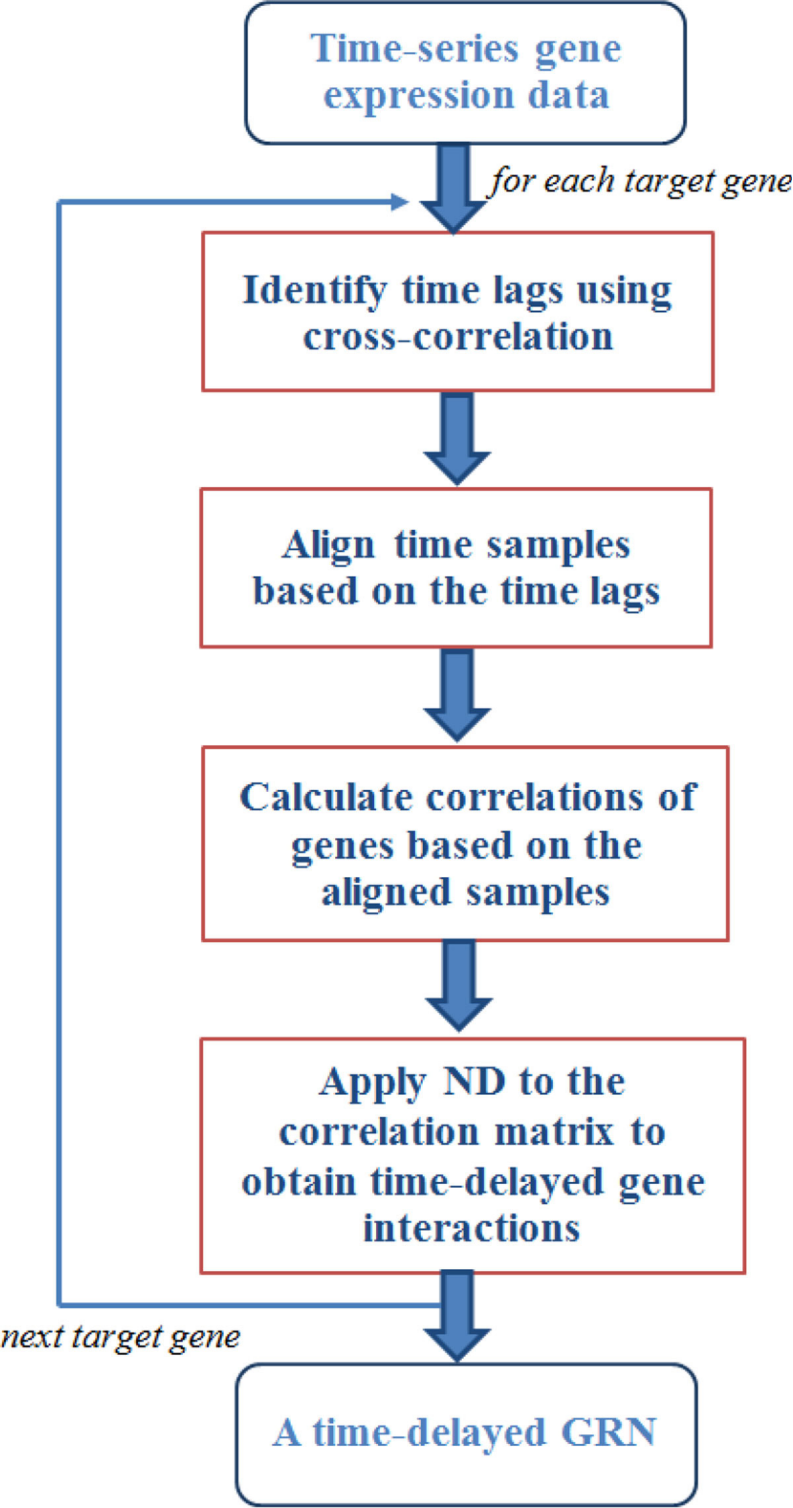
**The flow chart of time-delayed ND**.

We incorporate time delays into network deconvolution to enhance its strength in GRN inference. The flow chart of time-delayed ND is shown in Figure 7. Starting with the time-series gene expression data, for each target gene we perform the following steps. We first identify the time lags of regulation based on cross-correlation. Then we align the time samples based on the inferred time lags. After that, we calculate the correlations for each gene pairs based on the aligned samples, and apply ND on the correlation matrix. Finally, we obtain a GRN with time-delayed regulation.

Algorithm 1 shows more technical details about the procedure of time-delayed ND. For each gene in the set of *N *genes, the same process is carried out, which consists of three steps. First, with a fixed maximum time delay *r*, we identify the most likely time delays with the maximum absolute cross-correlation for the interaction between the target gene and each of the other genes. Second, we align the time samples for the target gene based on the time delays (see Algorithm 2), and compute correlations between this gene and the other genes based on the aligned samples. Third, we apply ND on the matrix of correlations and obtain a new matrix with direct dependencies among genes. Then the direct dependencies between the target gene and the other genes are extracted and stored. In this way, we infer time-delayed regulation among genes from time-series gene expression data.

**Figure 8:**
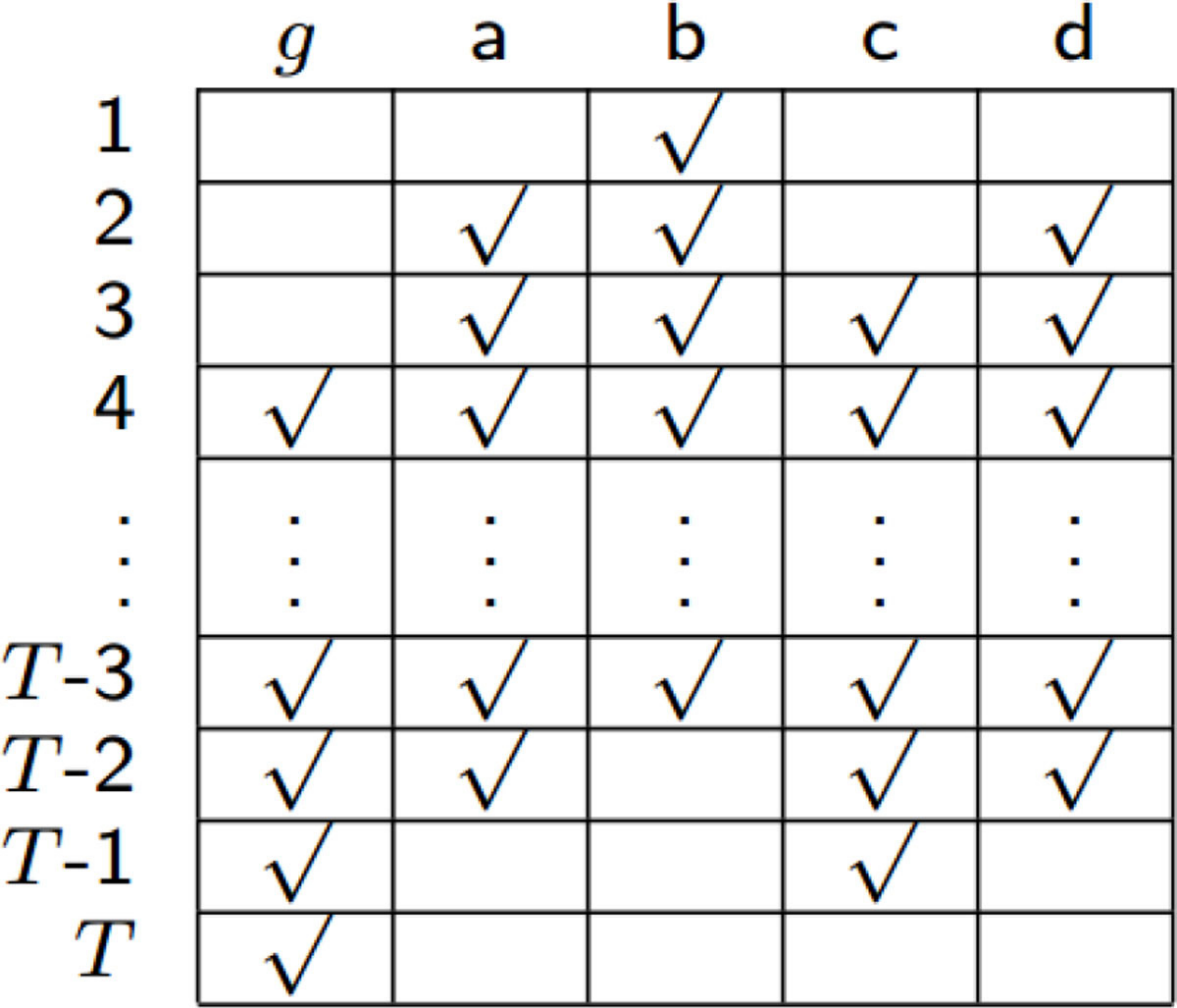
**Alignment of time samples based on time lags**. Time samples labeled with √ are used for calculating correlations after time lags being determined (here *l_ga _*= 2, *l_gb _*= 3, *l_gc _*= 1, *l_gd _*= 2).

In aligning time samples based on the inferred time lags, we assume that the time-series data are not periodic. Recall that *r *is the maximum order of regulation between the target gene and its regulators. Then effectively we have *T − r *samples based on which we calculate the correlations between the target gene and its possible regulators. The procedure of aligning time samples is presented in Algorithm 2. Figure 8 is an example showing how to do the alignment of time samples based on the lags between the target gene *g *and its possible regulators (gene *a, b, c *or *d*). The symbol *√ *inside a slot indicates that the corresponding time sample will be used to calculate the correlation, while the empty slots mean that those samples will not be used to calculate the correlations between this target gene and its potential regulators. When the time-series data are periodic, a similar method of alignment can be used, except that in this case we can use all the *T *time samples, in which the time points are shifted circularly [6].

### Evaluation

The performance of methods is evaluated using *Sensitivity, Precision *and *F-measure*. We define "positive" as the presence of a connection, and "negative" as the absence of an edge. The numbers of true positives, true negatives, false positives, and false negatives are denoted as *TP, TN, FP*, and *FN*, respectively. Then *Sensitivity *(denoted as *Se*; also known as *recall *), *Precision *(*P r*), *F-measure *(*Fm*), and *Specificity *(*Sp*) are defined as $Se=\frac{TP}{TP+FN}$, $Pr=\frac{TP}{TP+FP}$, $Fm=2*\frac{Pr*Se}{Pr+Se}$, $Sp=\frac{TN}{TN+FP}$. *F-measure *provides a balanced criterion to evaluate the performance of methods in GRN inference. A method with high *F-measure *implies that it can recover most true edges while most edges inferred by this method are correct. Here we use *F-measure *as the major criterion for comparing different methods.

**Algorithm 1**. Inferring time-delayed regulation based on Network Deconvolution

**INPUT**: Time-series gene expression data *X*_(*T ×N *) _with *N *genes and *T *time samples; the maximum possible time lag *r*

**OUTPUT**: An *N *× *N *matrix *S *with weights to show the strength of interactions among genes; An *N *× *N *matrix *D *with time-delayed information for each interaction

Normalize *X *so that the expression data for each gene have "zero" mean and "one" standard deviation

**for **each gene *i ***do**

Initialize a temporary vector $L_{i}^{*}=\left[\;\right]$

**for **each gene *j *in the rest genes **do**

Calculate the cross-correlation *C*(*x_i_, x_j_*) for gene *i *and gene *j *based on Eq. 3

Identify the time lag *l_ij _*with the maximum absolute cross-correlation *C*(*x_i_, x_j_*) among *r *choices of time lags

Store *l_ij _*into $L_{i}^{*}$

end for

Obtain the matrix $X_{i}^{\prime }$ with aligned time samples for target gene *i *according to Algorithm 2

Initialize a temporary *N *× *N *matrix *C^∗ ^*= [ ]

**for **each gene *i^′ ^***do**

**for **each gene *j^′ ^***do**

   Compute the correlation *corr_ij _*between gene *i′ *and gene *j′ *based on the corresponding time samples in $X_{i}^{\prime }$.

   Store *corr_ij _*into *C*^∗^

end for

end for

Apply ND on *C^∗ ^*to obtain *S^∗ ^*which denotes the direct interactions among genes based on the extracted time samples.

Extract the vector $S_{i}^{*}$ which contains the direct dependencies between gene *i *and its potential regulators and store it into *S, S *= [S $S_{i}^{*}$]

Append the values of time lags to *D, D *= [D $L_{i}^{*}$]

end for

Return *S *and *D*

**Algorithm 2**. Aligning time samples based on the delays inferred by cross-correlation

**INPUT**: Time-series gene expression data *X*_(*T ×N *) _; the matrix of time delays *L_i_^∗ ^*for target gene (denoted as the *i*-th gene); the maximum possible time lag *r*

**OUTPUT**: An (*T *− *r*) × *N *matrix $X_{i}^{\prime }$ with aligned time samples for the target gene

Initialize $X_{i}^{\prime }$ = [ ]

Extract the vector of time samples for the target gene (the *i*-th gene) from *X, y *= *X*(*r *+ 1 : *T, i*)

Append the expression of the target gene to $X_{i}^{\prime }$, $X_{i}^{\prime }$ = [$X_{i}^{\prime }$*y*]

**for **each gene *j *(*j ≠ i*) **do**

Find time delay *l_ij _*of interaction between the target gene *i *and gene *j *from $L_{i}^{*}$

Extract and align time samples for gene *j *from *X *based on *l_ij _, x_j _*= *X*(*r *− *l_ij _*+ 1 : *T *− *l_ij _, j*)

Append the expression of the *j*-th gene to $X_{i}^{\prime }$, $X_{i}^{\prime }$ = [$X_{i}^{\prime }$*x_j_*]

end for

Return $X_{i}^{\prime }$*^′^*

## Competing interests

The authors declare that they have no competing interests.

## Authors' contributions

H.C. developed algorithm and performed experiments. H.C., P.A. and J.Z. analyzed results, interpreted results, and wrote the manuscript. L.Z. helped in performing the experiments. J.Z. and F.L. provided overall supervision, direction and leadership to the research.

## Additional information

^1 ^The derivation of Eq. 2: Using the convolution expression, we have *ϕ_xy _*(*τ *) = *x*(−*τ *) ∗ *y*(*τ *); Converting *x*(−*τ *) to frequency domain using Fourier transform $FT\left[x\left(-\tau \right)\right]=\int_{-\infty }^{\infty }x\left(-\tau \right)\text{exp}\left(-i2\pi ft\right)d\tau$; Substituting *τ ^′ ^*= −*τ *we have $FT\left[x\left(-\tau \right)\right]=\int_{\infty }^{-\infty }-x\left(\tau^{\prime }\right)\text{exp}\left(i2\pi f\tau^{\prime }\right)d\tau^{\prime }=\int_{-\infty }^{\infty }x\left(\tau^{\prime }\right)\text{exp}\left(i2\pi f\tau^{\prime }\right)d\tau^{\prime }=X^{*}\left(f\right)$; Combining the equations above we have Φ*_xy _*= *FT*[*ϕ_xy _*(*τ *)] = *X*^∗ ^(*f*)*Y*(*f*); Applying inverse Fourier transform we can obtain *ϕ_xy _*as in Eq. 2.

## Declarations

The publication charges for this article were funded by AcRF Tier 1 Seed Fund on Complexity (RGC 2/13).

This article has been published as part of *BMC Systems Biology *Volume 8 Supplement 4, 2014: Thirteenth International Conference on Bioinformatics (InCoB2014): Systems Biology. The full contents of the supplement are available online at http://www.biomedcentral.com/bmcsystbiol/supplements/8/S4.
